# Trained Nurses and the Anglo-French Committee

**Published:** 1916-12-09

**Authors:** K. E. Kiero Watson

**Affiliations:** Matron. 83 Pall Mall, London, S.W.


### THE EDITOR'S LETTER-BOX.

### Trained Nurses and the Anglo-French Committee.

*To the Editor of* THE HOSPITAL.

SIR,—In reference to the memorandum recently issued to the effect that all trained nurses wishing to work under the Anglo-French Committee must first put their services at the disposal of the War Office for enrolment in the Army Nursing Service Reserve if required, it has now been arranged that instead of personally applying to the Matron-in-Chief at the War Office, they should apply to the Matron of the Anglo-French Committee, 83 Pall Mall, S.W., who will apply for the necessary sanction from the War Office.—Yours faithfully,

K. E. KIERO WATSON, Matron.

83 Pall Mall, London, S.W., December 5, 1916.

